# Supplementary material for: Prevalence of oncogenic human papillomavirus (HPV 16/18) infection, cervical lesions and its associated factors among women aged 21–49 years in Amhara region, Northern Ethiopia
Source: PLoS One. 2021 Mar 24;16(3):e0248949. doi: 10.1371/journal.pone.0248949 (PMC7990306; doi:10.1371/journal.pone.0248949)
Supplement: Attachment — Submitted filename: Response to Reviewers.docx [file pone.0248949.s002.docx]

**Response to the Editor and Reviewers:**

Dear Editor and Reviewers!

The authors would like to thank the area editor and the reviewers for their precious time and invaluable comments to our submitted article “Prevalence of oncogenic human papilloma virus (HPV 16/18) infection, cervical cancer and its associated factors among women aged 21-49 years in Amhara region, Northern Ethiopia”. We greatly appreciate the thorough and thoughtful comments provided to our paper. We are resubmitting the revised version according to the comments and we believe that the revised version of our paper addresses all concerns. Minor revisions and grammatical corrections are applied to the manuscript modification along with revisions explained in this document. Attached below are detailed responses to the comments. The comments are in black and the authors’ responses are in red.

**Response to the Editor**
1) It seems each VIA positive patient is considered as having cervical cancer. This doesn't appear to be accurate since VIA would be positive for pre-cancer lesions as well which the authors acknowledge at line 80. Please consider rephrasing the relevant text to include pre-cancer to VIA results for example at page 10 Line 168 and elsewhere

Response: It is addressed

- VIA can be positive in precancerous or cancerous lesions and therefore pre-cancer lesion is added to relevant section in the revised version.

2) Line 128 which variables were included in the multivariable logistic regression?

Response: We appreciate the query. Variables with p-value less than 0.2 in binary logistic regression were included in multivariable logistic regression. Based on this we have now mentioned as indicated in line 138 of the revised manuscript

- For association of HPV acquisition; age of first sexual intercourse, HIV status, occupation and marital status were included in multivariable logistic regression
- For association of VIA positivity; age, age of first sexual intercourse, HIV status, educational status, occupation, time of oral contraceptive use and marital status were included in multivariable logistic regression

3) Table 2 „Vaccinated for cc“ should be „Vaccinated for HPV“

Response: Corrected

4) Line 170 typo „HVP“ should be HPV

Response: Corrected

5) Line 174 and elsewhere please rephrase „abnormal cervical cytology“ to „cervical lesions“ or something similar since cytology was not performed

Response: Thank you for this important comment. We rephrase abnormal cervical cytology to cervical lesions

6) Tables 5 & 6 why were different variables considered for HPV or VIA positivity?

Response: The variables are selected based on the assumption and certain evidences that factors for acquiring HPV infection are different from factors for developing cervical cancer. Therefore, variables in table 5 (current age, age of first sexual intercourse, HIV status and history of sexually transmitted illness) are related to sexual behavior which could affect acquisition of HPV, whereas, variables in table 6 (age of first sexual intercourse, HIV status, residence, history of STI, educational status and parity) were considered as factors for cervical cancer.

7) Table 6 the numbers for „Residence“ do not add up to 337

Response: It is addressed

8) Sentence at line 223-224 is unclear. Possibly the authors meant „cervical cancer complaint“?

Response: It is addressed

9) Language and grammar should be somewhat improved

- It is done

**Response to Reviewer #1**

1. Title: “Prevalence of oncogenic human papillomavirus (HPV 16/18) infection, cervical cancer and its associated factors among women aged 21-49 years in Amhara region, Northern Ethiopia”. The “…oncogenic human papillomavirus (HPV 16/18) infection…” is one of the risk factors for cervical cancer. But it's independently or separately mentioned in the topic. In the topic; there also “… it's associated factors…” though this is not clear to which condition these factors are considered to be associated. So, the author would restructure the title as…; “Prevalence of cervical cancer and its associated factors…”.

Response: Thank you for this suggestion. As the reviewer points out, oncogenic human papilloma virus infection is one of the risk factor for cervical cancer; however our study was also aimed to describe the prevalence of HPV with associated factors for infection. By considering this, we believe the title as it is would be more explanatory.

2. Abstract: Methods: Line 25: They would discuss sample calculation, sampling techniques, method of analysis used to address the prevalence and associated factors.

Response: Thank you for this comment. It is addressed

- The sample calculation used was single population proportion formula and women were consecutively recruited (page 2, line 29, line111). Sixteen percent HPV prevalence, 5% margin of error and 1.5 design effect was considered for the calculation. (Line 111)

3. Abstract: conclusion: line 41 to 43: “…This study also identifies early age sexual contact, high parity and being uneducated/low educational status as primary risk factors to the development of cervical cancer…”. The study design of this study was “cross-sectional”, but the author tried to conclude the finding as “risk factors”. The risk factor is not a good expression or not appropriate for such a study design. Therefore; the author would replace phrases like “…associated factors”. See the topic.

Response: It is addressed.

- Risk factor is replaced with associated factor

4. Introduction: page: Line 75-78: “…In the Amhara region, testing for HPV infection has been not available or if available it is in limited areas, and hence little is known about the prevalence of HPV infection and cervical cancer, their co-prevalence’s and factors contributing to HPV infection and progression to cervical cancer”. The author mentioned the lack of HPV tests in the region as a justification to conduct the current study. This is not a good reason to conduct this study because the study has nothing to do with the availability or lack of HPV test facilities. Surprisingly; the study is conducted where the HPV test facilities are available. Can the author respond to this question?

Response: We sincerely appreciate the comment. The reviewer mention lack of HPV test or availability in limited area cannot be justification to conduct the current study.

- We would like to mean due in many health facilities of the region, the prevalence remain unknown (line 84) and also indicated in the method. The current study provided HPV test kit for the study purpose to selected hospitals which have HPV testing setups (testing machine). Although the study has nothing to do (or plan to do) with the availability of HPV tests in health facilities, by revealing the facts about the prevalence and associated factors, it recommend to scale up HPV testing to every facility in the region.

5. Methods and materials: Study setting: Line 81: the author would briefly discuss the information the study settings that address health care system/health services in the region (health coverage, No.hospital, No. health center, coverage of cervical cancer care centers, HPV tests facilities, etc.)

Response: We acknowledge this information pointed out by the reviewer

- We added description of the study setting, the health care system and cervical cancer services in the region to the study setting ( Line 95)

6. Methods and materials: Study setting: Line 86-90: “All women who came for routine gynecologic or family planning services to those hospitals were used as a source population for this study. Women 88 age 21 to 49 years and referred to the cervical cancer screening services during the study period were included in the study. However, women with known cervical cancer and pregnancy were excluded”. This statement is talking about the study population and misplaced here. Therefore; the author would move it to the “study population section”.

Response: It is addressed and moved to study population (Line 101

7. Methods and materials: Sample size and sampling technique: Line 91: the authors should clarify how they draw/select the participants.

Response: It is addressed.

- The sample size is calculated as indicated in comment 2 and study participants were recruited consecutively

8. Methods and materials: data collection: Line 98: the interview technique must be clarified. Was it at the entry point or exit point?

Response: We apologize for our lack of clarity. It is clarified in the revised version (Line 115).

- All eligible clients were screened for cervical lesions using VIA and tested for HPV. Study participants were recruited at the exit point for interview and review of medical records to get VIA and HPV results after getting informed consent.

9. Result part: How did the authors handle the relationship between HPV and cervical cancer?

Response: It is an established fact that HPV infection is a risk factor for cervical cancer. In our study we focused on describing the prevalence of oncogenic HPV and cervical cancer or precancerous lesions separately with possible associated factors for HPV acquisition and cervical lesions, thus HPV and cervical cancer association was not described.

10. Result: Table 5 (line 191), table 6 (line 201): “bivariate logistic regression…” what does it mean by “bivariate logistic regression”?. Since you are doing with multiple factors affecting the outcome variable?

Response: We thank the reviewer for pointing out the type of analysis in relation to factors affecting the outcome variable. We have considered the comment and both crude odds ratio (COR) and adjusted odds ratio (AOR) was used to describe the association in table 5 (Line 215) and table 6 (Line 246)

11. Discussion: Line 213: “…The observed HPV prevalence in the present study is lower compared to studies in another part of Ethiopia, Gurage zone, 17.3%[12], and Atta hospital of rural Ethiopia, 16% [7]. Also, the prevalence of presumed cervical cancer is lower than studies reported from southern Ethiopia 16.5% [13]”. The authors tried to justify reasons as “…Knowledge about the prevalence of HPV among women with normal and abnormal cervical cytology is important to monitor and design HPV control programs…”. What does it mean? Did you explored the knowledge of women in this regard? If “yes”, was it high or low? Comment: the author would discuss the implication for health policy and practices. E.g the lower prevalence in your finding may be due to the inaccessibility of the services.

Response: The sentence “…Knowledge about the prevalence of HPV….” was intended for description of knowledge about HPV among women with and without cervical lesion and it is another concept not put to justify the low prevalence of HPV, thus was deleted for its ambiguity and the reason for low prevalence is justified; variation in detection methods ( in line 260), exclusion of pregnant women and cervical cancer cases (264).

**Response to Reviewer #2**

Comments
Remove ‘this’ line 79

Response:- It is done

Q1, Please, clearly explain the inclusion and exclusion criteria. Line 87-90, “women age 21-49 years referred to the cervical cancer screening services during the study…”
Was this cervical screening service for the purpose of study or it was given as a routine service at all the four included hospitals?
Did the participant send it to the cervical cancer screening test after consent or before?
where was consent taken? Was at ANC, PMTCT, FP, or at the cancer screening center?

Response: Thank you for pointing this out.

- Cervical screening service was given as a routine service in all hospitals. But for the purpose of the study HPV test kit was provided and all eligible clients screened for cervical lesions were also sent to test for HPV. Study participants were recruited at the exit point, after getting the service (VIA and HPV test). Consent was sought for interview and review of medical records to get VIA and HPV and HIV results. (Data collection: Line 115).
- The inclusion criteria: Women of age 21 to 49 years eligible for cervical cancer screening visiting the clinics for routine services during the study period were included (Line 104)
- Exclusion criteria: women with known cervical cancer and pregnancy were excluded (line 106).

Q2, Ethical considerations, and consent procedures should be included in the body of the manuscript.

Response: It is done (Line 333)

Q3, Participants also screened for HIV. What does screening mean? Were the participants tested for HIV? Or asked for self-reporting their HIV status? If tested, did they counseled? who performed the testing? How did you manage refusals?

Response: HIV testing is part of cervical cancer screening program and the result is obtained from medical records. The ethical issue was addressed in the consent.

Q4, why did you use a design effect in this study? The design effect is used for multistage sampling studies. But this study is a facility-based cross-sectional study.

Response: Thank you for this comment. Since the hospitals are found in different zonal administrations of the region, they are considered as clusters. Therefore, even though the study was cross sectional, design effect was used.

Q5, Modify table name for table 1. – “Demographic characteristics of study participants, women aged 21-49 years…”,. The Demographic characteristics are not about the whole Amahar region. Follow an appropriate table naming and apply this for all other tables.

Response: It is done

Q6, Remove the total raw end of table 1. It is confusing and it is better to put n=337 at the headers of the table as Frequency(N= 337). Apply this for all other tables.

Response: it is done

Q7, Line 151, replace “had not heard” by never heard about

Response: It is done

Q8, Line 224: “visiting family planning or gynecology clinics 224 were excluded from the present study” Was this included in the exclusion criteria? And justify the reason why the women were excluded.

Response: We apologize for our lack of clarity. The exclude ones were women of cervical compliant visiting the clinics. It is corrected
