# Supplementary material for: Prevalence of oncogenic human papillomavirus (HPV 16/18) infection, cervical lesions and its associated factors among women aged 21–49 years in Amhara region, Northern Ethiopia
Source: PLoS One. 2021 Mar 24;16(3):e0248949. doi: 10.1371/journal.pone.0248949 (PMC7990306; doi:10.1371/journal.pone.0248949)
Supplement: Attachment — Submitted filename: Response to Reviewers.docx [file pone.0248949.s003.docx]

**Response to the Reviewers:**

Dear Reviewers!

The authors would like to thank the reviewers for their precious time and vital comments to our submitted article titled “**Prevalence of oncogenic human papilloma virus (HPV 16/18) infection, cervical lesions and its associated factors among women aged 21-49 years in Amhara region, Northern Ethiopia**” (PONE-D-20-26948R1). We greatly appreciate the comments and we are resubmitting the revised version. We believe the revised version addresses all concerns and attached below are detailed responses to the comments.

**Response to Reviewer #2**

1. Abstract page 2, Line 29-31, there is a repetition of the whole sentence

Response: - sorry for the repetition, It is corrected

2. Line 94: Avoid spacing before comma

Response: Thank you for this comment. It is addressed

3. Line 73-74 Revise the sentence ending with “in all most all cervical cancer cases”.

Response: It is addressed and corrected as “in almost all cervical cancer cases”

4. Line 351-499: Reference writing (font and spacing)

Response:- Thank you, the font and spacing is corrected

**Response to Reviewer #3**

- Cervical cancer prevention programs in Ethiopia and the status of VIA, HPV testing and vaccination in Ethiopia and Amhara region should be elaborated. In addition, low number of enrolled women is major limitation

Response: Thank you for this point.

The content of cervical cancer prevention and control program in Ethiopia is included in the introduction based on 2015 Ethiopian Ministry of health cervical cancer prevention and control guideline in Line 80, Line 87-96 of the revised manuscript and the low sample size is written in the limitation of the study.

- Abstract Method line 23-27, unnecessary repetition

Response: It is addressed

- Introduction line 64 “and late detection of HPV infection” should be “and late detection of cervical lesion”

Response: It is corrected

- Introduction line 86 what does it stand for “cervical cancer prevention program”

Response: Thank you for this comment. It is elaborated based on the local context.

- Introduction line 79: “where HPV testing is unavailable” should be “where cervical cancer screening by cervical cytology and HPV testing is unavailable”

Response: It is done

- Result: all numbers should be in one decimal

Response: it is done

- Discussion: when referring to others studies be precise, Author et al. When comparing your method of testing to others be precise as well and cite the appropriate reference

Response: It is addressed

**Response to Reviewer #4**

The focus of the study article is very important to Ethiopia and other developing countries still using VIA as a cervical cancer screening strategy. However, the uptake of VIA is low and there are a lot of issues associated with its sensitivity and specificity. Besides, quality control is another grey area in the implementation of VIA as a primary screening strategy. Onco6 HPV antigen test is one of the HPV tests available in the market with high specificity for lesions associated with HPV 16 and 18 but its sensitivity is low as a test and this is the inherent problem of the test. As close to 70% of cervical cancer is associated with HPV16 and 18 the test is assumed to be one of the good options in the provision of HPV based cervical cancer screening service. However, as the purpose of cervical cancer screening is to identify women with pre-cancerous lesion and viruses other than HPV 16 and 18 maybe important in the study settings thus this particular study will miss significant number of high-risk HPV infections. Thus, with the test type employed the authors will be able to identify only persistent HPV16 and 18 infection and not the prevalence of high-risk HPV and this needs to be corrected in their text. Although VIA is advocated for its specificity by those who recommend the test for primary screening, VIA missed seven out of 24 Onco6 test positives and 27 of VIA positives were Onco6 test negatives. Of course, about seven of the 27 Onco6 test negatives had STI history. So many factors account for the progression of the HPV infection to cervical cancer, HPV genotype is one and they were only able to identify HPV 16 and 18 and not others by the method they have employed. As cervical cancer is a kind of progressive disease identifying factors associated with progression of HPV infection cannot be met by such correctional study design. In summary, the finding of this study is publishable if the authors rather show how VIA is neither a sensitive nor a specific test to identify pre-cancerous lesions. The authors need further to look at their data justify why 61% (27/44) of the VIA positive were Onco6 negative and recommend further study to assess the utility of VIA to identify specific pre-cancerous lesions among those who are Onco6 test positives.

Response: Thank you for pointing out issues related to

- persistent HPV16 and 18 infection versus high-risk HPV
- the uptake, sensitivity and quality control of VIA,
- the importance of HPV genotypes other than 16 and 18,
- persistent HPV 16 and HPV 18 infection versus prevalence of high-risk HPV

The sensitivity and specificity of VIA was calculated and added to the result section (line 213-216). VIA identified 17 and missed 7 out of 24 HPV positive women that make its sensitivity 70.8% and 286 out of 293 participant women were correctly identified negative which make VIA specificity 91.3%. However, the positive predictive value of VIA is only 61.4% (27 out of 44). The low sensitivity of VIA is indicated in the limitation and further study is recommended (Line 348)

Regarding the HPV genotypes our study aimed to HPV 16 and HPV 18 which are considered highly prevalent genotypes associated with cervical cancer. Since women visiting gynecology and family planning service were recruited for the study, it is believed to be prevalence.
